# Establishment of a novel double-antibody sandwich fluorescence microsphere immunochromatographic test strip for rapid detection of swine acute diarrhea syndrome coronavirus (SADS-CoV) infection

**DOI:** 10.3389/fcimb.2025.1461845

**Published:** 2025-02-28

**Authors:** Xiao Cong, Fei Tong, Huizhen Liu, Yujun Zhu, Ningxin Tan, Feng Gu, Huanan Wang, Feng Cong

**Affiliations:** ^1^ Guangdong Laboratory Animals Monitoring Institute, Guangdong Provincial Key Laboratory of Laboratory Animals, Guangzhou, China; ^2^ Guangdong Provincial Key Laboratory of Organ Donation and Transplant Immunology, the First Affiliated Hospital, Sun Yat-sen University, Guangzhou, China; ^3^ Fu Shun Vocational Technology Institute, Fushun, China; ^4^ College of Coastal Agricultural Sciences, Guangdong Ocean University, Zhanjiang, China; ^5^ Ministry of Agriculture (MOA) Key Laboratory of Animal Virology, Center for Veterinary Sciences, Zhejiang University, Hangzhou, China

**Keywords:** swine acute diarrhea syndrome coronavirus (SADS-CoV), fluorescent microsphere-based immunochromatography assay (FM-ICA), monoclonal antibody (mAb), point-of-care testing (POCT), antigen

## Abstract

**Introduction:**

Swine acute diarrhea syndrome coronavirus (SADS-CoV) is an enveloped, positive-sense, single-stranded RNA virus that causes clinical symptoms such as vomiting and diarrhea in 10-day-old piglets. SADS-CoV has caused significant economic losses in the swine industry in southern China. Currently, no effective treatments or vaccines are available for this disease, making it crucial to establish a point-of-care testing (POCT) technology for early diagnosis and prevention.

**Methods:**

In this study, we first validated the specificity and immunogenicity of four monoclonal antibodies (mAbs) targeting the nucleocapsid (N) protein of swine acute diarrhea syndrome coronavirus (SADS-CoV). The optimal antibody pair for constructing the fluorescent microsphere-based immunochromatographic assay (FM-ICA) was determined through systematic pairwise screening. Critical parameters of the FM-ICA test strip, including antibody labeling concentration, coating concentration, incubation time, and sample dilution ratio, were subsequently optimized. Analytical performance characteristics of the developed FM-ICA were then rigorously evaluated. Finally, clinical validation was conducted by parallel testing of 72 field samples using both FM-ICA and quantitative PCR (qPCR), followed by concordance rate analysis.

**Results:**

First, we demonstrated that all four monoclonal antibodies exhibited favorable immunogenicity and specificity. Subsequently, mAb 12E1 was identified as the coating antibody, and mAb 5G12 was selected as the labeled antibody, forming the optimal combination for FM-ICA preparation. After optimization, the ideal parameters were determined: a labeling concentration of 200 μg/mg for antibodies, a coating concentration of 1 mg/mL, an incubation time of 10 min, and a dilution factor of 10. The FM-ICA exhibited outstanding specificity, sensitivity, reproducibility, and stability, achieving a maximum detectable dilution factor of 1280 and a limit of detection (LOD) of 78 PFU mL⁻¹. Finally, the concordance rate between FM-ICA and qPCR for clinical samples reached 97.22%.

**Discussion:**

These results indicate that FM-ICA is an excellent POCT technology that can be used for the early diagnosis of SADS-CoV, providing support for disease prevention and treatment.

## 1 Introduction

Coronaviruses, a type of positive-sense, single-stranded RNA virus, belong to the order Nidovirales, family Coronaviridae, and genus Coronaviruses. There are four genera within the coronavirus family: alpha, beta, gamma, and delta. Coronaviruses cause respiratory and gastrointestinal diseases in humans (Hu et al., 2021), mammals (Zhou et al., 2018), and birds (Woo et al., 2012). At present, six coronaviruses are known to cause diseases in pigs: transmissible gastroenteritis virus (TGEV), porcine respiratory coronavirus (PRCV), porcine epidemic diarrhea virus (PEDV), swine acute diarrhea syndrome coronavirus (SADS-CoV), porcine hemagglutinating encephalomyelitis virus (PHEV), and porcine delta coronavirus (PDCoV) (Wang et al., 2019). It is worth noting that some of these viruses have been discovered recently.

In 2017, the first outbreak of swine acute diarrhea syndrome (SADS) occurred in the southern region of China (Yang et al., 2020). This disease causes clinical symptoms such as vomiting and diarrhea and has a high mortality rate of up to 90% in piglets (Pan et al., 2017). Within a span of just 4 months, approximately 25,000 piglets died in the affected area, resulting in significant economic losses for local farms (Zhou et al., 2018). The causative agent of this disease was SADS-CoV, an alpha coronavirus. The complete genome of this virus is approximately 2.7 kb in length and encompasses four structural proteins (spike [S], envelope [E], membrane [M], and nucleocapsid [N]) as well as various non-structural and accessory proteins. Among them, the nucleocapsid protein is highly conserved and can be used as a target protein for qualitative detection of the virus (Cong et al., 2023).

Fluorescent microspheres (FMs) are small, spherical particles with diameters typically ranging from 10 nm to 1 µm. They consist of fluorescent dyes or fluorescent proteins encapsulated within polymer or glass materials, forming tiny spherical particles. FMs have wide applications in the fields of cell imaging, bioanalysis, and diagnostics (Ji et al., 2020). Fluorescent microsphere-based immunochromatography assay (FM-ICA) technology is an innovative immunological detection technique that employs fluorescent microspheres as labels conjugated with antibodies or antigens for biomolecule detection and analysis. The carboxyl groups on the surface of fluorescent microspheres can be stably coupled to the amino groups on the surface of antibodies via the reaction with 1-ethyl-3-(3-dimethylaminopropyl) carbodiimide (EDC)/N-hydroxysuccinimide (NHS), ensuring their secure attachment and preventing detachment. Currently, there is still a need to develop new technologies for detecting SADS-CoV to achieve disease prevention goals. Therefore, the FM lateral flow assay technique established in this study holds significant importance for the prevention and detection of SADS-CoV.

## 2 Materials and methods

### 2.1 Virus, antibodies, samples and main reagents

Clinical samples, including feces and intestinal contents, were collected from breeding farms in southern China in accordance with the recommendations of the National Standards for Laboratory Animals of the People’s Republic of China (GB149258-2010). These samples were stored in the New Detection Technology Center of the Guangdong Laboratory Animal Monitoring Institute.

In this study, nine porcine viruses, including classical swine fever virus (CSFV), porcine reproductive and respiratory syndrome virus (PRRSV), pseudo rabies virus (PRV), swine influenza virus (SIV), Seneca valley virus (SVA), transmissible gastroenteritis virus (TGEV), porcine epidemic diarrhea virus (PEDV), porcine delta coronavirus (PDCoV), and swine acute diarrhea syndrome coronavirus (SADS-CoV), were prepared in the laboratory (Cong et al., 2022).

The preparation process of the monoclonal antibody mAb 5G12 was previously published in an article (Cong et al., 2023). The other three monoclonal antibodies (mAb 1B1, 2H6 and 12E1) were obtained from subsequent research in our laboratory. For the specific preparation methods, please refer to the articles published by our laboratory (Cong et al., 2023).

Goat anti-Mouse IgG (GAM) was purchased from Beyotime Biotechnology (Shanghai, China). The FMs, 1-ethyl-3-(3-dimethylaminopropyl) carbodiimide (EDC), N-hydroxysuccinimide (NHS), bovine serum albumin (BSA), and 2-(N-morpholino) ethanesulfonic acid (MES) buffer were purchased from SIGMA (USA). The polyvinyl chloride (PVC) plates, nitrocellulose (NC) membranes, sample pads, and absorbent pad were purchased from Millipore (USA).

### 2.2 Coupling of antibodies with fluorescent microspheres

The specific operation steps were as follows: (1) the microspheres in the whole tube were vibrated with a vortex shaker for 1 min, and the microspheres precipitated at the bottom of the centrifugal tube were dispersed in the liquid to obtain a uniform microsphere suspension. (2) An appropriate amount of microsphere suspension was taken into a 1.5 mL centrifuge tube and centrifuged at 8000×g for 2 min, before aspirating the supernatant with a pipette gun. (3) 100 µL ddH_2_O was added, vortexed for 1 min and centrifuged at 8000×g for 2 min, before aspirating the supernatant with a pipette gun. (4) 1 mL 0.01 M sodium dihydrogen phosphate solution (NaH_2_PO_4_) with pH 6.0 was added, and the ultrasonic homogenizer was used to clean the microspheres. (5) After centrifuging at 8000×g for 2 min, the supernatant was aspirated with a pipette gun. (6) 1 mL of activation solution (10 mg EDC and 6 mg NHS dissolved in 1 mL of 0.01 M NaH_2_PO_4_) was added to the tube to resuspend the microspheres, before vortexing for 15 min. (7) step (5) was repeated. (8) 100 µL 50 mM pH 5.0 MES was added into the activated microspheres, and the ultrasonic homogenizer was used to resuspend the sediment. Then, 100-300 µg antibodies were added into the activated microspheres as labeled antibodies. (9) the mixture was placed on a shaker, incubated at room temperature for 2 h and centrifuged at 8000×g for 2-3 min, before aspirating the supernatant with a pipette gun. (10) 1 mL blocking solution (pH 7.4 0.01 M PBS, 1% BSA) was added, vortexed for 1 min, incubated on the shaker at room temperature for 1 h and centrifuged at 8000×g for 2-3 min, before aspirating the supernatant with a pipette gun. Subsequently, the tube was washed twice with phosphate-buffered saline containing 0.05% Tween 20 (PBST). (11) 1 mL of storage solution (pH 7.4 0.01 M phosphate-buffered saline [PBS], 1% BSA and 0.1% sodium azide] was added to the tube and mixed well to obtain the microsphere-antibodies conjugate.

### 2.3 Fabrication of the immunochromatographic test strip

**Figure 1:**
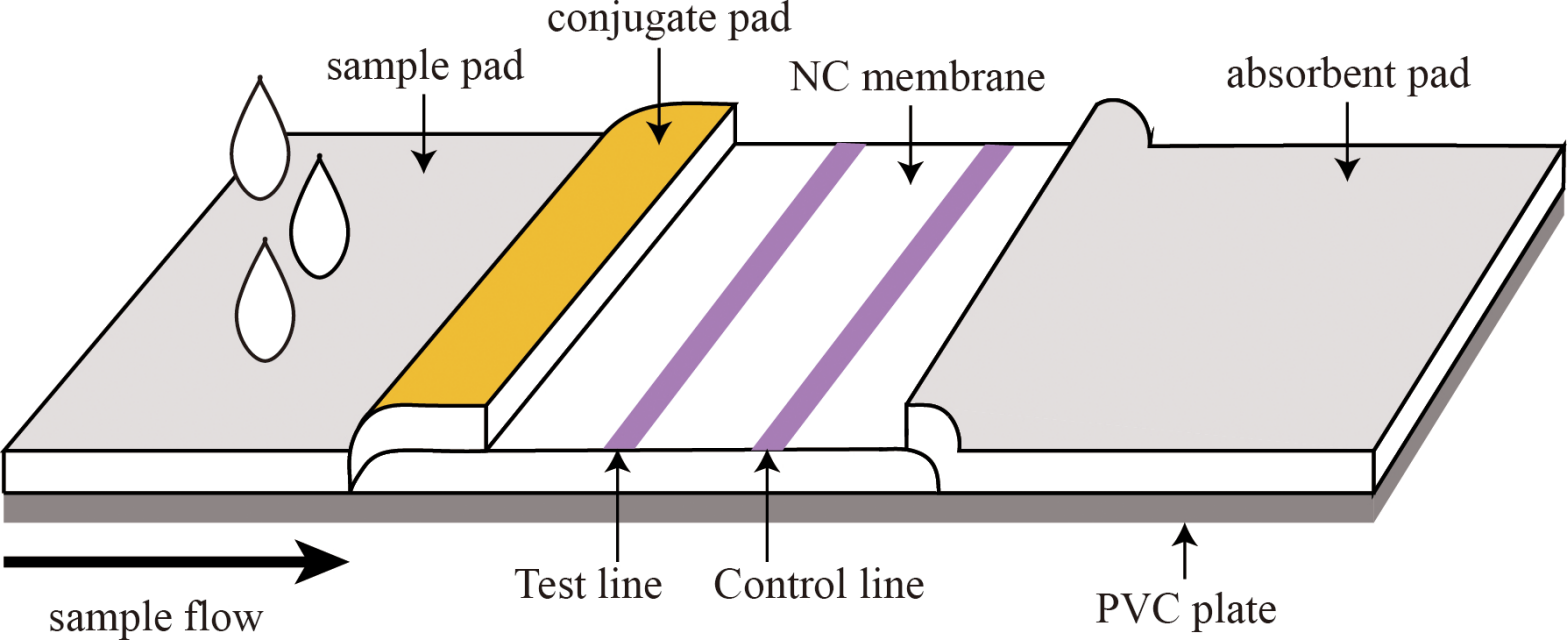
Structure of the fluorescent microsphere-based immunochromatography assay (FM-ICA) test strip.

As shown in **Figure 1**, the immunochromatographic test strip consisted of the following parts: a sample pad, conjugate pad, NC membrane, absorbent pad, and PVC plate. To prepare the conjugate pad, the microsphere-antibody conjugate was employed, and the conjugate pad was saturated with the labeled antibody using either a spraying or impregnating technique. To prepare the test line (T line) and the control line (C line), coated antibodies (T line) and GAM IgG (C line) were immobilized on the NC membrane by contact deposition at a dispensing speed of 6 µL/cm using an autodispensor (Biodot, USA). The as-prepared conjugate pad and NC membrane were dried at 37°C. The sample pad, conjugate pad, NC membrane, and absorbent pad were stacked on the PVC backing plate in turn, with a 2-mm overlap and cut into 3.5-mm-wide strips using a guillotine cutter (Fenghang, China) for further use (Deng et al., 2022).

### 2.4 Pairwise screening of monoclonal antibodies

In this study, the matrix method was employed to conduct pairwise screening experiments using four previously prepared monoclonal antibodies (mAb 5G12, mAb 1B1, mAb 2H6, and mAb 12E1) targeting the N protein. Each antibody was used as either a capture or label, and its binding with the other three monoclonal antibodies was tested. The capture concentration was set at 1 mg/mL, and the labeling amount was 200 µg/mg (indicating 1 mg of fluorescent microspheres conjugates to every 200 µg of antibody). The dilution factor of sample was ten, the incubation time was 10 min, and the optimal antibody combination was determined based on the fluorescence ratio value between the T and C lines (H_T_/H_C_) for subsequent experiments.

### 2.5 Optimization of the test strip

After selecting the optimal antibody combination, systematic optimization was performed for the conjugate complex, antibody capture concentration, sample dilution, and chromatography time.

### 2.6 Sample preparation

The SADS-CoV viral solution (MOI of 3) was diluted in maintenance medium (trypsin concentration of 8 µg/ml) at a ratio of 1:100. The diluted virus solution was then filtered through a 0.22-µm membrane for subsequent experiments. Vero cells were cultured until reaching 80%–90% confluency, at which point the culture medium was discarded. The cells were washed twice with PBS buffer, and then the filtered and diluted virus solution was added to cover the entire cell monolayer. The cells were incubated in a cell culture incubator for 2 h, before adding maintenance medium. The cytopathic effect (CPE) of cells was observed after 48 h. When CPE of cell fusion occurred, the cells were lysed, and the virus was collected.

Viral RNA was extracted using an automatic nucleic acid extraction and purification instrument (Beyotime Biotechnology, China), and the early pre-synthesized primers (F: 5′-CGCGGATCCATGGCCACTGTTAATTGGGGTGACGCT-3′ and R: 5′-CCGCTCGAGCTA-ATTAATAATCTCATCCACCATCTC-3′) targeting the SADS-CoV N gene (GenBank: MK651076.1 (Cong et al., 2023), 1128bp) were used for PCR amplified to identify the virus.

Before being dropped onto the sample pad of the FM - ICA, virus samples and clinical samples need to be lysed with a lysis buffer. The composition of the lysis buffer is: 20 mM Tris - HCl (pH 7.6), 137 mM NaCl, 1% Triton X - 100, 10% glycerol (optional, for protein stabilization), and 5 mM EDTA. This lysis buffer can ensure the rupture of the viral envelope, release the protein contents, and maintain protein homeostasis simultaneously.

### 2.7 Western blot, indirect immunofluorescence assay (IFA) and confocal microscopy

Vero cells were cultivated in 96-well cell culture plates and inoculated with SADS-CoV when the cells reached 80%~90% density. When a CPE appeared, the plates were washed three times with PBS. The cells were fixed using precooled 4% paraformaldehyde for 30 min at 37°C and then blocked with 1% BSA at 37°C for 2 h. After blocking, the cells were incubated with mAbs (1:2000 dilution) for 1 h, before incubating with fluorescein isothiocyanate (FITC)-conjugated goat anti-mouse antibody (1:100 dilution) for 1 h at 37°C. Fluorescent images were collected using a confocal laser scanning microscope (Zeiss, JENA, Germany). For the specific experimental procedures of Western blot, please refer to the articles published by our laboratory (Cong et al., 2023).

### 2.8 Functional analysis of FM-ICA

Under the optimal conditions, the performance of the developed test strip was evaluated in terms of its sensitivity, specificity, stability, and repeatability.

Nine viruses, including classical swine fever virus (CSFV), porcine reproductive and respiratory syndrome virus (PRRSV), pseudo rabies virus (PRV), swine influenza virus (SIV), Seneca valley virus (SVA), transmissible gastroenteritis virus (TGEV), porcine epidemic diarrhea virus (PEDV), porcine delta coronavirus (PDCoV), and swine acute diarrhea syndrome coronavirus (SADS-CoV), were evaluated to investigate the specificity of the detection card. PBS buffer was used as a negative control. The nine viruses were subjected to a 10-fold dilution reaction, and the negativity or positivity of the reaction was determined by observing the H_T_/H_C_ values.

PBS was used as the sample diluent, and positive samples were diluted in serial two-fold dilutions, with dilution factors of 2, 5, 10, 20, 40, 80, 160, 320, 640, 1280, 2560, and 5120. PBS buffer was used as the negative control, and the detection limit of detection (LOD) was assessed on the basis of the H_T_/H_C_ values.

To validate the stability of the detection card, the prepared test strips were individually placed in a 37°C incubator for 7, 14, 21, and 28 days. Chromatographic reactions were performed at the corresponding time intervals.

Finally, to assess the repeatability of the detection card, two dilutions (10 and 160 dilution) of the antigen were replicated eight times each, and the coefficient of variation was calculated.

### 2.9 FM-ICA for the detection of clinical samples

A total of 72 swine clinical samples, consisting of 24 intestinal luminal content and 48 fecal samples from healthy piglets and piglets with diarrhea symptoms (these piglets are less than 15 days of age), were detected using the SADS-CoV FM-ICA testing card. These results were compared with qRT–PCR to check for any nonspecific amplification. Finally, the conformity rate of the detection results was assessed to determine whether it has clinical application value.

## 3 Results

### 3.1 Cultivation of viruses and validation of monoclonal antibodies

**Figure 2:**
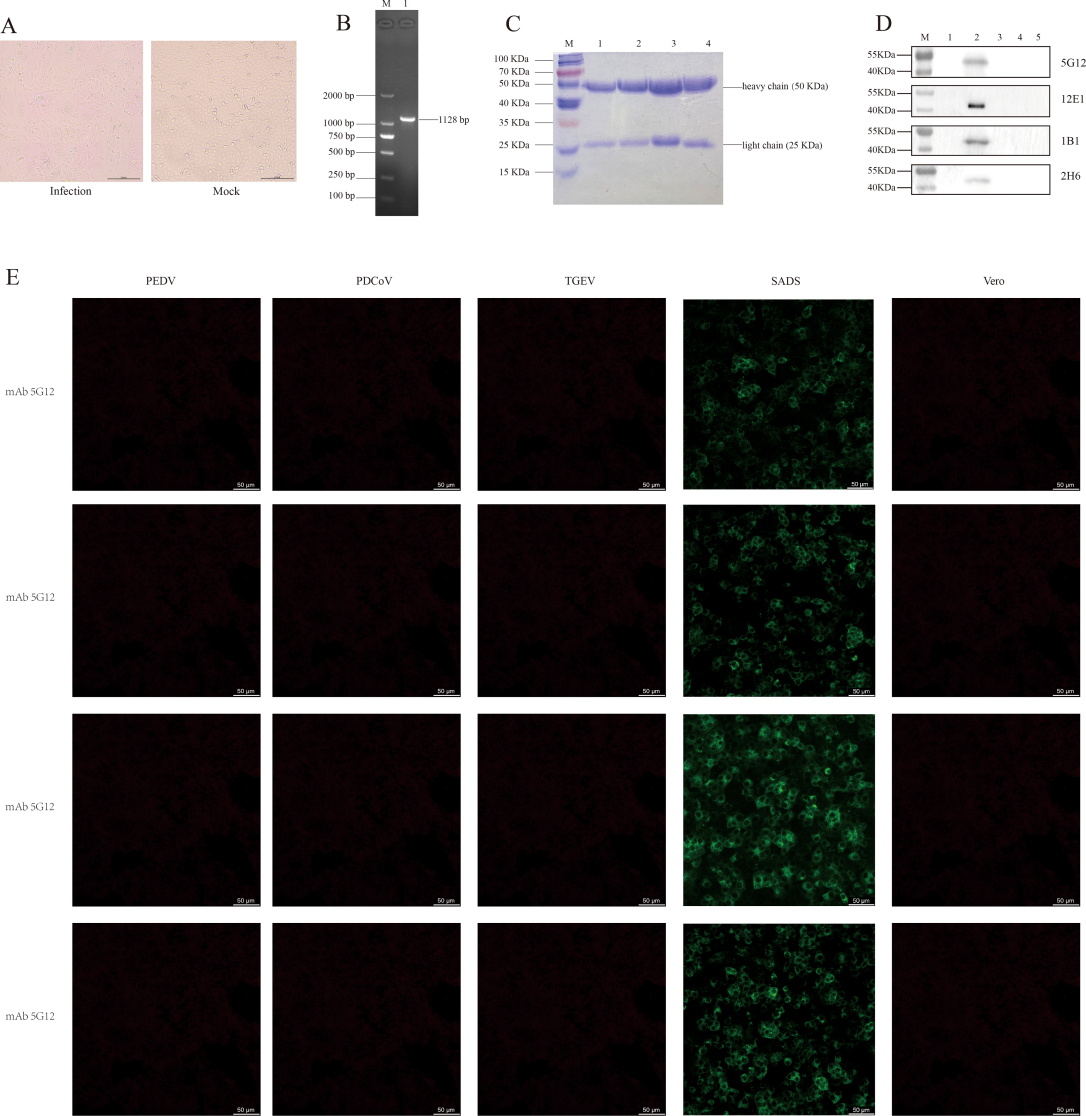
Cultivation and identification of SADS-CoV virus, and verification of four monoclonal antibodies. **(A)** CPE of SADS-CoV (left, 200 µm); Vero cells not infected with the virus (right, 200 µm). **(B)** PCR identification of the SADS-CoV N gene, with an amplified product of approximately 1128 bp. M, Marker; line 1, N gene. **(C)** SDS-PAGE results of the four monoclonal antibodies. M, Marker; line 1, mAb 5G12; line 2, mAb 1B1; line 3, mAb 2H6; line 4, mAb 12E1. **(D)** Western blot results of the four mAbs reacting with the whole virus. M, Marker; line 1, Vero cells; line 2, SADS-CoV whole virus; line 3, TGEV whole virus; line 4, PEDV whole virus; line 5, PDCoV whole virus. **(E)** IFA results of the four mAbs reacting with PEDV, PDCoV, TGEV and SADS-CoV (50 µm).

Vero cells exhibited CPE approximately 48 h after SADS-CoV inoculation, as shown in **Figure 2A**, left). The observed syncytial cell morphology at this stage is characteristic of the virus’s typical pathological changes. The PCR identification results also confirmed the aforementioned conclusion (**Figure 2B**). And through preliminary validation, the Multiplicity of Infection (MOI) of the virus was determined to be 10^5^ PFU mL^-1^.

The four monoclonal antibodies used in this study were validated by SDS-PAGE. As evident from the results in **Figure 2C**, all four monoclonal antibodies exhibited two distinct bands, representing the heavy chain (50 KDa) and light chain (25 KDa), which is a typical characteristic of monoclonal antibodies. This also indicates that these monoclonal antibodies are of high purity. Furthermore, the results from both IFA and western blotting demonstrated that all four monoclonal antibodies exhibited good specificity and immunoreactivity, specifically binding to the SADS-CoV N protein (approximate size of 42 KDa), without binding to other porcine coronaviruses such as TGEV, PEDV, and PDCoV (**Figures 2D, E**).

### 3.2 Pairwise screening of monoclonal antibodies

**Figure 3:**
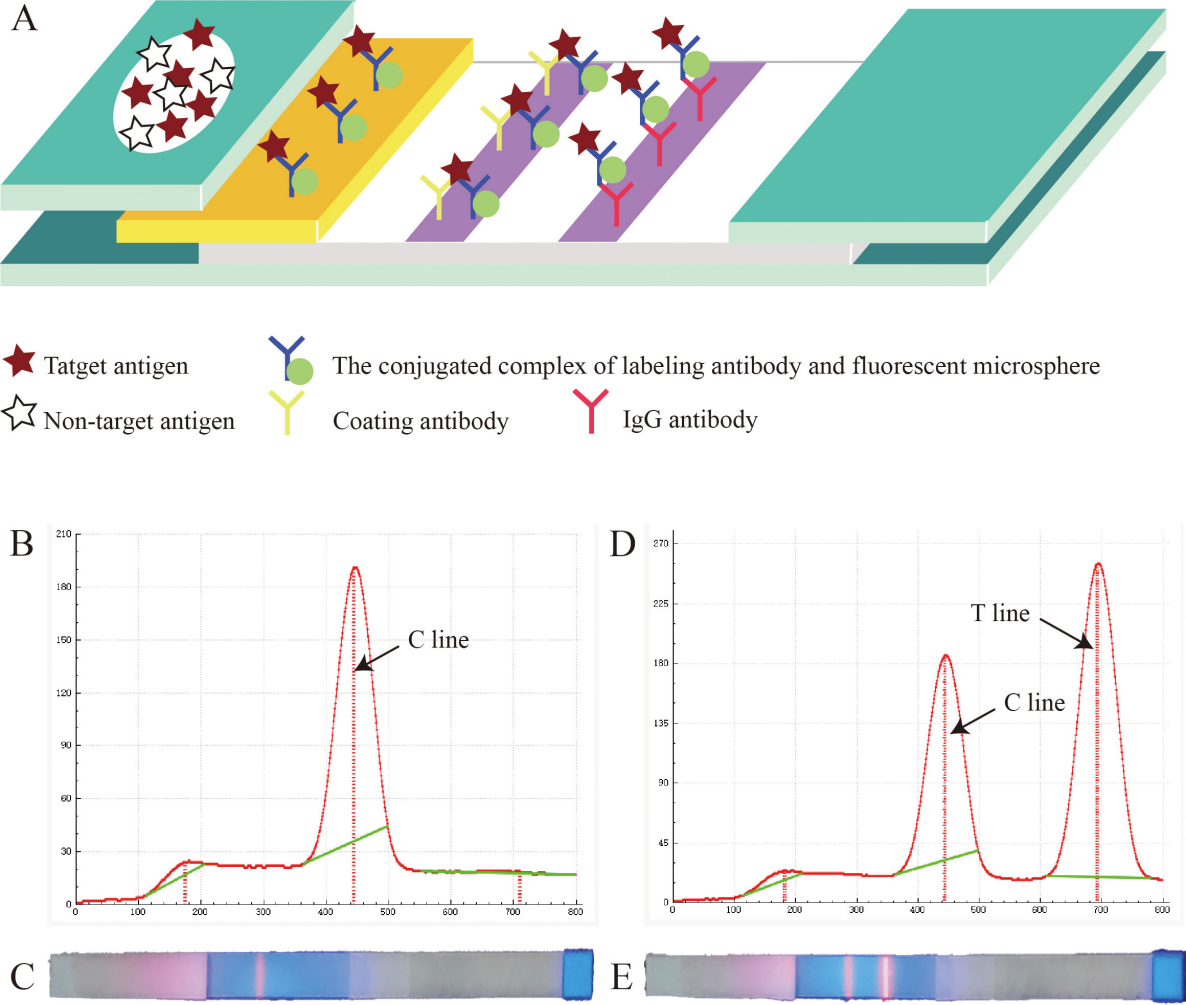
Principle of the fluorescent microsphere-based immunochromatography assay (FM-ICA) test strip. **(A)** Reaction schematic diagram of FM-ICA. **(B)** Fluorescence reading graph of the negative sample. **(C)** The results of the negative sample under ultraviolet light. **(D)** Fluorescence reading graph of the positive sample. **(E)** The results of the positive sample under ultraviolet light.

The working principle of this test strip is shown in **Figure 3A**. Briefly, when the sample solution is added onto the sample pad, it slowly migrates toward the absorbent pad by capillary action. As the sample passes through the conjugate pad, the conjugate complex containing FMs binds to the target antigen, forming a new “antigen-antibody-FM” complex. Subsequently, this complex binds to the coating antibodies at the T line, emitting fluorescence. The IgG antibodies on the C line bind to the conjugate complex on the conjugate pad, emitting fluorescence, thereby confirming the proper execution of the detection process. As shown in **Figure 3B**, **C**, for negative samples, no fluorescence signal could be observed at the T line, whereas the C line could be read with a normal fluorescence value. As shown **Figures 3D, E**, for positive samples, both the T and C lines exhibited detectable fluorescent signals, and the positivity of the sample was determined by assessing the fluorescence ratio (H_T_/H_C_). Interestingly, if the requirements for virus quantification are not strict, the results can be directly observed with the naked eye using an ultraviolet flashlight (365 - nm fluorescence ultraviolet) (**Figures 3C, E**).

Based on this principle, we used the matrix method, the four monoclonal antibodies were individually coated or labeled. As shown in **Table 1**, the H_T_/H_C_ ratio was the highest when mAb 12E1 was used as the coating antibody and mAb 5G12 as the labeling antibody. Therefore, this antibody pair was selected as the optimal combination for further studies.

**Table 1:** H_T_/H_C_ ratio of FM-ICA in monoclonal antibody pairwise screening.

Labeling antibody name (200µg/mg)	Coating antibody name (1mg/mL)											
	5G12			1B1			2H6			12E1		
5G12	/	/	/	0.33	0.41	0.37	0.27	0.24	0.23	1.29	1.32	1.36
1B1	0.29	0.26	0.56	/	/	/	0.39	0.42	0.42	0.15	0.26	0.28
2H6	0.12	0.15	0.26	0.16	0.28	0.31	/	/	/	0.49	0.59	0.62
12E1	0.13	0.22	0.27	0.49	0.57	0.62	0.22	0.28	0.33	/	/	/

### 3.3 Optimization of the FM-ICA test strip

After choosing mAb 12E1 as the coating antibody and mAb 5G12 as the labeling antibody, other parameters, such as the optimal antibody labeling amount, optimal antibody coating concentration, and optimal chromatography duration, were optimized.

**Figure 4:**
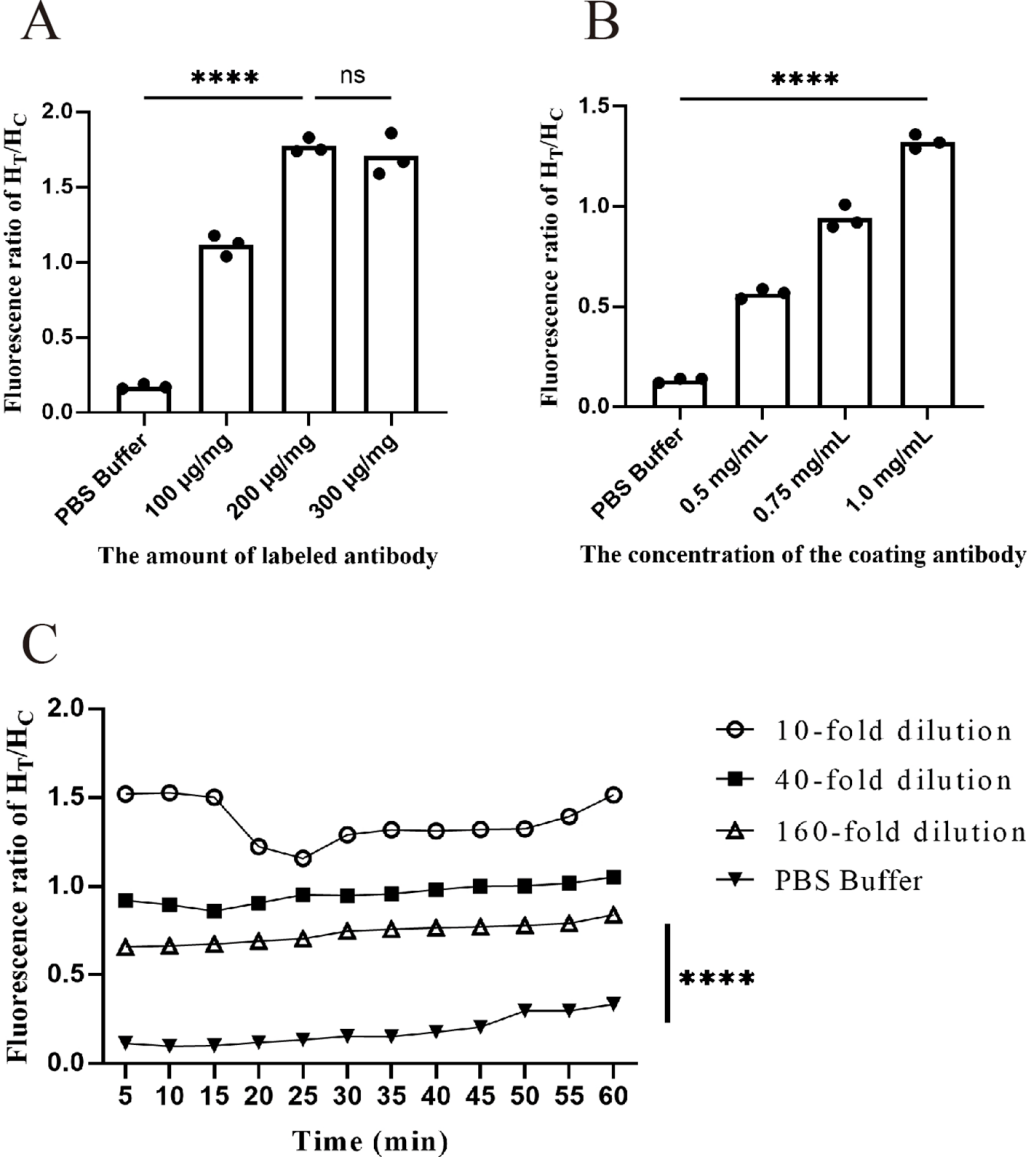
Optimization of FM-ICA system conditions. **(A)** Fluorescence ratio when different numbers of antibodies were coupled to each milliliter of fluorescent microspheres. **(B)** Fluorescence ratio at different antibody coating concentrations. **(C)** Fluorescence ratio of samples with three dilution factors at 5-min intervals within 60 min. **(A, B)** During the optimization of labeling and coating concentrations, the samples were all diluted 10-fold, n = 3. (****p < 0.0001; ns, not significant).

Because of the limitation of carboxyl groups on the surface of FMs, the conjugation of antibodies with FMs can reach saturation after a certain quantity. Therefore, it is necessary to determine the minimum saturation number of 1 mg FMs to achieve optimal results while minimizing material waste. As shown in **Figure 4A** (**Supplementary Table 1**), when the coating antibody concentration was fixed at 1 mg/mL, the addition of 200 µg of antibody resulted in saturation of the FM conjugation. The H_T_/H_C_ ratio reached its peak value (1.77). However, when 300 µg of antibody was conjugated to 1 mg of fluorescent microspheres, there was no significant difference in the fluorescence ratio. Therefore, 200 µg/mg was chosen as the optimal antibody labeling amount for FM-ICA.

We next fixed the labeling antibody amount at 200 µg/mg and continued to explore the optimal coating antibody concentration. As evident from the results in **Figure 4B** (**Supplementary Table 2**), within a certain range, the fluorescence ratio showed an antibody concentration-dependent increase. Therefore, when the coating antibody concentration was 1 mg/mL, the ratio was the highest and significantly different from that of the control. Hence, 1 mg/mL was chosen as the optimal coating antibody concentration.

After determining the optimal coating and labeling concentrations, we investigated the optimal sample chromatography time for the test strip. Fluorescence readings were taken every 5 min within the 5–60 min timeframe for three sample dilutions: high (10-fold dilution), medium (40-fold dilution), and low (160-fold dilution). The H_T_/H_C_ ratio was calculated for each time point. As shown in **Figure 4C** (**Supplementary Table 3**), at a dilution factor of 10, the fluorescence ratio peaked at 10 min. However, beyond the 15-min mark, the ratio became unstable and declined. Similarly, in the PBS buffer group, an H_T_/H_C_ ratio greater than 0.3 was observed after 45 min, indicating a false positive phenomenon. These findings demonstrate that prolonged time has a significant impact on fluorescence values. Taking all factors into consideration, a chromatography time of 10 min was selected as the optimal reading period.

### 3.4 Analytical performances of the test strip

**Figure 5:**
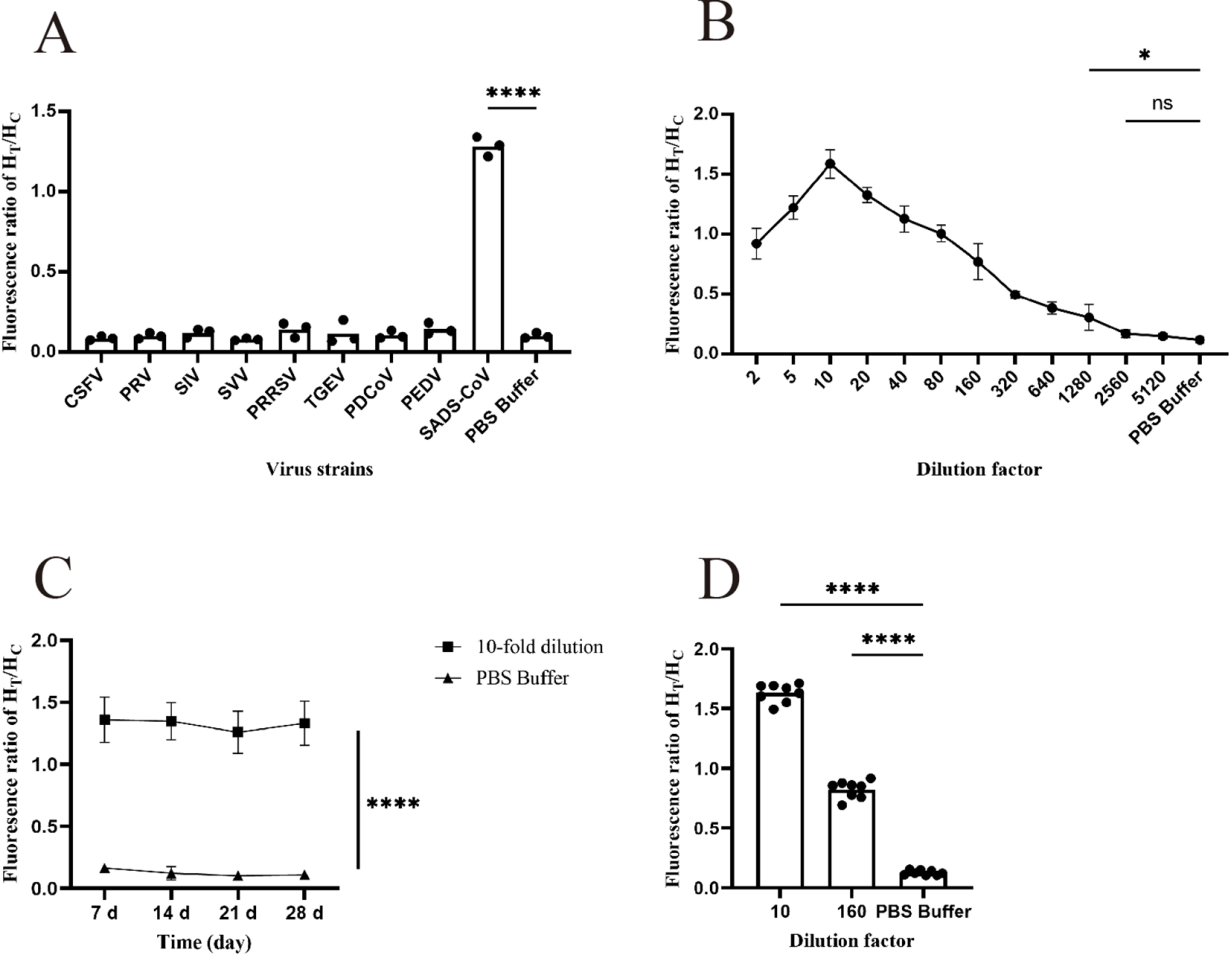
Functional validation of the prepared FM-ICA. **(A)** The specificity of FM-ICA was verified using nine porcine-related viruses (n = 3). **(B)** The detection limit of FM-ICA was determined by serially diluting the samples (n = 3). **(C)** The stability of FM-ICA was evaluated using samples diluted 10-fold (n = 3). **(D)** The reproducibility of FM-ICA was assessed using samples diluted at two dilution factors, (n = 8) (*p < 0.05 and ****p < 0.0001; ns, not significant).

Specificity testing is an essential step in the development of any detection method because good specificity is a fundamental requirement for establishing a detection method. In this study, nine pig-related viruses, including CSFV, PRRSV, SIV, PRV, SVV, TGEV, PDCoV, PEDV, and SADS-CoV, were used for specificity analysis. As shown in **Figure 5A** (**Supplementary Table 4**), only SADS-CoV exhibited a fluorescence ratio of > 0.3, indicating a positive result, and the comparison with other viruses was statistically significant. Therefore, it is demonstrated that the test strip has good specificity.

Sensitivity is an indicator that evaluates the ability of a detection method to sensitively differentiate between different concentrations of viruses. In this study, the prepared samples were serially diluted with dilution factors of 2, 5, 10, 20, 40, 80, 160, 320, 640, 1280, 2560, and 5120 to explore the detection limit of FM-ICA. **Figure 5B** (**Supplementary Table 5**) demonstrates that when the sample was diluted 2560 times (LOD is 39 PFU mL^-1^), the H_T_/H_C_ ratio did not show statistical significance. However, when the sample was diluted 1280 times (LOD is 78 PFU mL^-1^), the result was statistically significant. Therefore, 78 PFU mL^-1^ was established as the LOD of FM-ICA. In addition, when the sample was diluted to 1280-fold, the H_T_/H_C_ ratio was approximately 0.30. In this study, H_T_/H_C_ > 0.30 was established as the threshold for determining a positive outcome. Similarly, **Figure 5B**, demonstrates that when the sample was diluted 10 times, the H_T_/H_C_ ratio reached its peak, whereas the fluorescence ratios decreased after dilutions of 5 and 20 times. Hence, it can be concluded that the optimal dilution factor for the samples in this study is 10.

The stability testing of FM-ICA mainly focuses on whether the test strips exhibit abnormal detection results after being stored for a certain time. Therefore, we stored the test strips in an incubator at 37°C for 7, 14, 21, and 28 days to observe the stability of the detection card under different storage times. As shown in **Figure 5C** (**Supplementary Table 6**), the fluorescence ratios of FM-ICA were not significantly differences under different storage times, indicating good stability of the detection card.

In this study, the antigens were diluted 10- and 160-fold, and each dilution gradient was repeated eight times. The coefficient of variation was calculated for both sets of results to assess the reproducibility of the detection method. The coefficient of variation (C.V.) is a normalized measure of the degree of dispersion in a probability distribution, which is calculated using the formula C.V. = SD/mean. In general, a smaller coefficient of variation indicates less dispersion in the dataset. In statistical analysis, if the coefficient of variation exceeds 15%, it is important to be cautious as it may indicate the presence of abnormal values, and consideration should be given to excluding such data points. Through calculations, the coefficients of variation for the 10-fold diluted antigen, 160-fold diluted antigen, and control groups in this study were found to be 4.39%, 8.40%, and 14.57%, respectively (**Figure 5D**; **Supplementary Table 7**). All coefficients of variation were below 15%. Therefore, it can be concluded that the FM-ICA established in this study exhibits good operational repeatability.

### 3.5 Clinical application of FM-ICA

To further validate the practicality of FM-ICA for on-site sample testing, 72 clinical samples provided by the Guangdong Laboratory Animals Monitoring Institute were assessed. These samples were collected from multiple pig farms in Guangdong Province and originated from 15-day-old piglets. The samples included both sick piglets with symptoms of diarrhea and vomiting as well as healthy piglets, comprising 24 intestinal luminal contents samples and 48 fecal samples. Initially, 200 µl of each sample was extracted for nucleic acid extraction, followed by reverse transcription and fluorescence quantitative PCR detection to qualitatively analyze the samples based on the Ct values (the primers and probe sequences are provided in **Table 2**; GenBank: MK651076.1). Subsequently, these samples were tested using the FM-ICA established in this study. Finally, the results from both detection methods were compared.

**Table 2:** Primers and probe sequences used for qRT–PCR detection of clinical samples.

Name	Sequences
SADS-qPCR-F	GATCAGCCTTCTAACTGGCACT
SADS-qPCR-R	CAAGACCTGTGGGGCTAGTT
SADS-qPCR-P	ACTGGTCCTCACGCAGATGCTCCT

**Figure 6:**
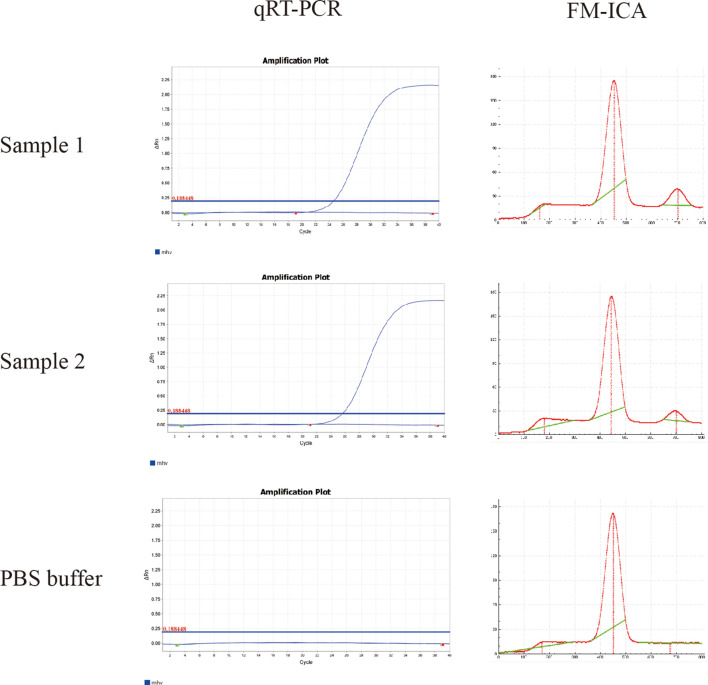
qRT-PCR and FM-ICA detection results of the two samples with inconsistent test results.

The comparative results are shown in **Table 3**, where FM-ICA detected nine positive samples, whereas qRT–PCR detected 11 positive samples. This indicates that there were two samples with inconsistent results between the two methods. Both of these samples had Ct values of 24.76 and 25.83, respectively, as detected by qRT–PCR. The fluorescence ratio values obtained from FM-ICA were 0.27 and 0.18, respectively. **Figure 6** demonstrates that the T line of FM-ICA exhibits weak fluorescence, but it cannot be considered positive. In contrast, qRT–PCR results with Ct < 30 can be determined as positive or weakly positive. These findings confirm that the sensitivity of FM-ICA in directly detecting samples is lower than that of qRT–PCR for nucleic acid detection. By calculation, the concordance rate (CR) for positive samples was 81.82% (9/11), the CR for negative samples was 96.83% (61/63), and the overall CR was 97.22% (70/72). This also indicates that the proposed method does not exhibit false-positive phenomena. Overall, the CR between the developed detection method FM-ICA in this study and qRT–PCR is greater than 90%, indicating that the method holds significant potential for clinical application.

**Table 3:** Comparison between the FM-ICA and qRT-PCR for detection of SADS-CoV.

		qRT-PCR			CR (%)
		+	-	total	
FM-ICA	+	9	0	9	97.22
	–	2	61	63	
	total	11	61	72	

CR, Concordance rate; Overall CR = (true positive + true negative)/total * 100%; +, positive sample; -, negative sample; Positive CR = FM-ICA positive/qRT–PCR positive * 100%; Negative CR = qRT–PCR negative/FM-ICA negative * 100%.

## 4 Discussion

In recent years, coronaviruses have shown a new trend of spread and re-emergence. The SADS-CoV mentioned in this study is similar to COVID-19 in that it is also a virus of bat origin, with a high nucleotide sequence similarity of up to 98.9% to the bat coronavirus HKU-2 strain (Li et al., 2018). Because of the significant threat it poses to the health of humans and livestock, it has attracted extensive attention from the academic community both domestically and internationally. There are currently four known types of porcine epidemic diarrhea viruses, namely SADS-CoV, PEDV, PDCoV, and TGEV. SADS-CoV exhibits clinical symptoms that are extremely similar to those of the other three diseases, making it difficult to differentiate in clinical practice. Moreover, no effective treatment methods or vaccines are currently available for this disease (Liu and Wang, 2021). Therefore, establishing a rapid detection method for SADS-CoV is crucial for early prevention and timely isolation.

Currently, methods for detecting SADS-CoV mainly involve nucleic acid, antigen and antibody detection. The nucleic acid detection method for SADS-CoV primarily relies on the design of specific primers or probes targeting the conserved regions of the SADS-CoV N or M genes. For instance, in previous research conducted by our team, a constant temperature nucleic acid amplification method called recombinase polymerase amplification (RPA) was developed using the M gene of SADS-CoV. This method has a detection limit of 74 copies/µL for SADS-CoV and a high concordance rate of 98.61% compared to fluorescence quantification. It enables rapid clinical detection of SADS-CoV (Cong et al., 2022). In a similar context, researchers have combined microfluidic chips with loop-mediated isothermal amplification (LAMP) technology to develop a novel detection method for the M gene of SADS-CoV. This method allows simultaneous detection of three viruses (PDCoV, PEDV, and SADS-CoV), offering characteristics such as accuracy, sensitivity, repeatability, and high specificity (Zhou et al., 2020). Furthermore, Liu developed a specific primer based on clustered regularly interspaced short palindromic repeats (CRISPR/Cas12a) and LAMP technology targeting PEDV ORF3, TGEV N, PDCoV N, and SADS-CoV N. They established a fluorescence-enhanced visual nucleic acid detection technique labeled with ROX, enabling rapid naked-eye identification of the four viruses. This method is highly suitable for fast detection in pig farms (Liu et al., 2022). Several PCR methods are available for SADS-CoV nucleic acid detection, such as droplet digital PCR (Zhang et al., 2022), multiplex PCR (Si et al., 2021; Niu et al., 2022; Zhou et al., 2022; Zhu et al., 2022), and TaqMan probe/SYBR green-based qPCR (Zhou et al., 2018; Ma et al., 2019; Pan et al., 2020).

Unlike nucleic acid testing, antibody detection methods for SADS-CoV primarily rely on the construction of recombinant proteins. Researchers fused immunoglobulin G (IgG) to the Fc end of the SADS-CoV S protein to construct an S-Fc recombinant protein. Based on this protein, they developed a simplified ELISA method for detecting SADS-CoV antibodies, which also exhibits good specificity and high sensitivity (Peng et al., 2022).

The targets of antigen-antibody testing and nucleic acid testing differ. Nucleic acid testing primarily focuses on the virus’s nucleic acids, antibody testing detects the antibodies produced by the body in response to the antigen, and antigen testing utilizes known antibodies to detect viral proteins. There is also a significant difference in the timing of antigen and antibody detection following a viral infection. Antigens can typically be detected within 1 to 3 days after infection, while the production of antibodies usually takes some time. Generally, IgM antibodies (early antibodies) may begin to appear around 5 to 7 days post-infection, whereas IgG antibodies (late antibodies) usually become detectable after 2 weeks or longer. Therefore, antigen testing is primarily used for early diagnosis and rapid screening, allowing for quicker determination of infection status and timely prevention. This is of significant importance for disease prevention. On the other hand, antibody testing helps to understand an individual’s immune status and evaluate the effectiveness of immunity. Currently, research on SADS-CoV antigen testing is limited. In 2023, researchers developed a method for detecting SADS-CoV antigen using a double-antibody sandwich assay with SADS-CoV antibodies. The principle of this method involves detecting the antigen using HRP-labeled antibodies (indirect ELISA) (Cao et al., 2023). While this approach shares a similar objective with our study, the key difference lies in our utilization of fluorescent microspheres to label antibodies, which are then fabricated into a test strip. This innovation significantly simplifies and accelerates the disease detection process, yielding results in as little as 10 minutes, thereby greatly optimizing the detection system. Moreover, our team is the first to employ fluorescent microsphere-conjugated antibodies in a double-antibody sandwich assay for the detection of SADS-CoV antigen.

The developed FM-ICA exhibits enhanced sensitivity compared to traditional colloidal gold test strips, enabling the detection of antigens even at low concentrations. Similar to the colloidal gold method, the method established in this study can also be visually observed within 10 minutes. However, the colloidal gold method doesn’t require any auxiliary tools, while the FM - ICA needs to use a 365 - nm fluorescent flashlight (the cost is approximately around 5 dollars). Different from the colloidal gold method, the method we established is much more sensitive and can be used for rough quantitative analysis within a certain range. For example, when the fluorescence ratio is high, it can be determined that the viral load is high; when the fluorescence ratio is low, it indicates that the viral infection is not severe. At the same time, although FM - ICA is simple and sensitive, in the absence of fluorescence, it depends on specific fluorescence instruments for reading values (the cost is approximately around 500 dollars), which is not required for the colloidal gold detection method. However, the results obtained using instruments for measurement are more objective and sensitive, reducing subjective judgment by the naked eye. And the detection method eliminates the need for nucleic acid extraction and PCR, which typically require skilled personnel to perform. Instead, clinical samples can be directly broken using lysis buffer and added to the sample well. After a waiting period of 10 minutes, the machine can direct display concentration level, enabling the assessment of viral replication within the animal. Therefore, this approach offers the advantage of high automation, simplifying the testing process significantly. Moreover, the detection cards are cost-effective, making this method highly suitable for high-throughput testing in pig farms.

In conclusion, the FM-ICA developed in this study is a detection technology that exhibits excellent stability, specificity, repeatability, and sensitivity. The concordance rate between clinical samples and qPCR detection results was 97.22%. These findings demonstrate that this method enables the rapid clinical detection of SADS-CoV, facilitating the timely prevention of epidemic outbreaks. This represents a highly promising novel POCT technology.

## Data Availability

The datasets presented in this study can be found in online repositories. The names of the repository/repositories and accession number(s) can be found in the article/**Supplementary Material**.

## References

[B1] Cao L. Kong X. Zhang Y. Suo X. Li X. Duan Y.. (2023). Development of a novel double-antibody sandwich quantitative ELISA for detecting SADS-CoV infection. Appl. Microbiol. Biotechnol. 107, 2413–2422. doi: 10.1007/s00253-023-12432-4 36809389 PMC9942060

[B2] Cong X. Zhu Y. Liu X. Lian Y. Huang B. Luo Y.. (2022). Establishment of a recombinase polymerase amplification (RPA) fluorescence assay for the detection of swine acute diarrhea syndrome coronavirus (SADS-CoV). BMC Vet. Res. 18, 369. doi: 10.1186/s12917-022-03465-4 36221092 PMC9552127

[B3] Cong X. Zhang L. Zhu H. Wu M. Zhu Y. Lian Y.. (2023). Preparation of a new monoclonal antibody against nucleocapsid protein of swine acute diarrhea syndrome coronavirus and identification of its linear antigenic epitope. Int. J. Biol. Macromolecules 239, 124241. doi: 10.1016/j.ijbiomac.2023.124241 36996959

[B4] Deng H. Chen D. Li X. Yang F. Liu S. Sun Y.. (2022). Development of a colloidal gold immunochromatographic test strip for the rapid detection of iprodione. Anal. Methods 14, 4370–4376. doi: 10.1039/D2AY01374F 36268701

[B5] Hu B. Guo H. Zhou P. Shi Z.-L. (2021). Characteristics of SARS-CoV-2 and COVID-19. Nat. Rev. Microbiol. 19, 141–154. doi: 10.1038/s41579-020-00459-7 33024307 PMC7537588

[B6] Ji C. Wei Y. Wang J. Zeng Y. Pan H. Liang G.. (2020). Development of a Dual Fluorescent Microsphere Immunological Assay for Detection of Pseudorabies Virus gE and gB IgG Antibodies. Viruses 12, 912. doi: 10.3390/v12090912 32825263 PMC7551494

[B7] Li K. Li H. Bi Z. Gu J. Gong W. Luo S.. (2018). Complete genome sequence of a novel swine acute diarrhea syndrome coronavirus, CH/FJWT/2018, isolated in Fujian, China, in 2018. Microbiol. Resour Announc 7, e01259–e01218. doi: 10.1128/mra.01259-18 30533848 PMC6284080

[B8] Liu J. Tao D. Chen X. Shen L. Zhu L. Xu B.. (2022). Detection of four porcine enteric coronaviruses using CRISPR-cas12a combined with multiplex reverse transcriptase loop-mediated isothermal amplification assay. Viruses 14, 833. doi: 10.3390/v14040833 35458562 PMC9032155

[B9] Liu Q. Wang H.-Y. (2021). Porcine enteric coronaviruses: an updated overview of the pathogenesis, prevalence, and diagnosis. Vet. Res. Commun. 45, 75–86. doi: 10.1007/s11259-021-09808-0 34251560 PMC8273569

[B10] Ma L. Zeng F. Cong F. Huang B. Huang R. Ma J.. (2019). Development of a SYBR green-based real-time RT-PCR assay for rapid detection of the emerging swine acute diarrhea syndrome coronavirus. J. Virol. Methods 265, 66–70. doi: 10.1016/j.jviromet.2018.12.010 30593837 PMC7113735

[B11] Niu J.-W. Li J. H. Guan J. L. Deng K. H. Wang X. W. Li G.. (2022). Development of a multiplex RT-PCR method for the detection of four porcine enteric coronaviruses. Front. Vet. Sci. 9, 1033864. doi: 10.3389/fvets.2022.1033864 36425116 PMC9679136

[B12] Pan Y. Tian X. Qin P. Wang B. Zhao P. Yang Y. L.. (2017). Discovery of a novel swine enteric alphacoronavirus (SeACoV) in southern China. Veterinary Microbiol. 211, 15–21. doi: 10.1016/j.vetmic.2017.09.020 PMC7117260 29102111

[B13] Pan Z. Lu J. Wang N. He W. T. Zhang L. Zhao W.. (2020). Development of a TaqMan-probe-based multiplex real-time PCR for the simultaneous detection of emerging and reemerging swine coronaviruses. Virulence 11, 707–718. doi: 10.1080/21505594.2020.1771980 32490723 PMC7549975

[B14] Peng P. Gao Y. Zhou Q. Jiang T. Zheng S. Huang M.. (2022). Development of an indirect ELISA for detecting swine acute diarrhoea syndrome coronavirus IgG antibodies based on a recombinant spike protein. Transbound Emerg. Dis. 69, 2065–2075. doi: 10.1111/tbed.14196 34148289

[B15] Si G. Niu J. Zhou X. Xie Y. Chen Z. Li G.. (2021). Use of dual priming oligonucleotide system-based multiplex RT-PCR assay to detect five diarrhea viruses in pig herds in South China. AMB Express 11, 99. doi: 10.1186/s13568-021-01255-z 34196816 PMC8246137

[B16] Wang Q. Vlasova A. N. Kenney S. P. Saif L. J. (2019). Emerging and re-emerging coronaviruses in pigs. Curr. Opin. Virol. 34, 39–49. doi: 10.1016/j.coviro.2018.12.001 30654269 PMC7102852

[B17] Woo P. C. Lau S. K. Lam C. S. Lau C. C. Tsang A. K. Lau J. H.. (2012). Discovery of seven novel Mammalian and avian coronaviruses in the genus deltacoronavirus supports bat coronaviruses as the gene source of alphacoronavirus and betacoronavirus and avian coronaviruses as the gene source of gammacoronavirus and deltacoronavirus. J. Virol. 86, 3995–4008. doi: 10.1128/JVI.06540-11 22278237 PMC3302495

[B18] Yang Y.-L. Yu J.-Q. Huang Y.-W. (2020). Swine enteric alphacoronavirus (swine acute diarrhea syndrome coronavirus): An update three years after its discovery. Virus Res. 285, 198024. doi: 10.1016/j.virusres.2020.198024 32482591 PMC7229464

[B19] Zhang Z. Wang N. Liu X. Lv J. Jing H. Yuan X.. (2022). A novel, reverse transcription, droplet digital PCR assay for the combined, sensitive detection of severe acute respiratory syndrome coronavirus 2 with swine acute diarrhea syndrome coronavirus. J. AOAC Int. 105, 1437–1446. doi: 10.1093/jaoacint/qsac039 35377440

[B20] Zhou H. Shi K. Long F. Zhao K. Feng S. Yin Y.. (2022). A quadruplex qRT-PCR for differential detection of four porcine enteric coronaviruses. Vet. Sci. 9, 634. doi: 10.3390/vetsci9110634 36423083 PMC9695440

[B21] Zhou L. Sun Y. Wu J. L. Mai K. J. Chen G. H. Wu Z. X.. (2018). Development of a TaqMan-based real-time RT-PCR assay for the detection of SADS-CoV associated with severe diarrhea disease in pigs. J. Virol. Methods 255, 66–70. doi: 10.1016/j.jviromet.2018.02.002 29427670 PMC7113665

[B22] Zhou L. Chen Y. Fang X. Liu Y. Du M. Lu X.. (2020). Microfluidic-RT-LAMP chip for the point-of-care detection of emerging and re-emerging enteric coronaviruses in swine. Anal. Chim. Acta 1125, 57–65. doi: 10.1016/j.aca.2020.05.034 32674781 PMC7234951

[B23] Zhou P. Fan H. Lan T. Yang X. L. Shi W. F. Zhang W.. (2018). Fatal swine acute diarrhoea syndrome caused by an HKU2-related coronavirus of bat origin. Nature 556, 255–258. doi: 10.1038/s41586-018-0010-9 29618817 PMC7094983

[B24] Zhu J.-H. Rawal G. Aljets E. Yim-Im W. Yang Y. L. Huang Y. W.. (2022). Development and clinical applications of a 5-plex real-time RT-PCR for swine enteric coronaviruses. Viruses 14, 1536. doi: 10.3390/v14071536 35891517 PMC9324624

